# Annales D’Oculistique

**Published:** 1895-04

**Authors:** 


ANNALES D'OCULISTIQUE, founded by Florent Cunier and continued by Warlomont. Edited by Dr. E. Valude and Dr. D. E. Sultzer, of Paris. The Trans-Atlantic Publishing Co. New York, 63 Fifth Ave.

This journal, originally published in French, is now published in English also and issued in New York. Dr. George T. Stevens is the editor of the English edition.

The present number is for January of 1895, and constitutes No. 1, of volume cxiii and the fifty-eighth year of its publication.

